# Supplementary material for: Intranasal cocaine self-administration in male mice
Source: Nat Commun. 2025 Dec 5;16:10916. doi: 10.1038/s41467-025-65875-w (PMC12680761; doi:10.1038/s41467-025-65875-w)
Supplement: Supplementary file 1 — Supplementary Information [file 41467_2025_65875_MOESM1_ESM.pdf]

Supplementary Materials for

Intranasal Cocaine Self-Administration in Mice

Kirsty R. Erickson, Yizhen Quan, Zahra Z. Farahbakhsh, Hannah E. Branthwaite, Keaton Song, Justin D. Kim, Janice J. Lee, Katherine N. Gibson-Corley, Eyal Y. Kimchi, Cody A. Siciliano*

*Corresponding Author Email: cody.siciliano@vanderbilt.edu

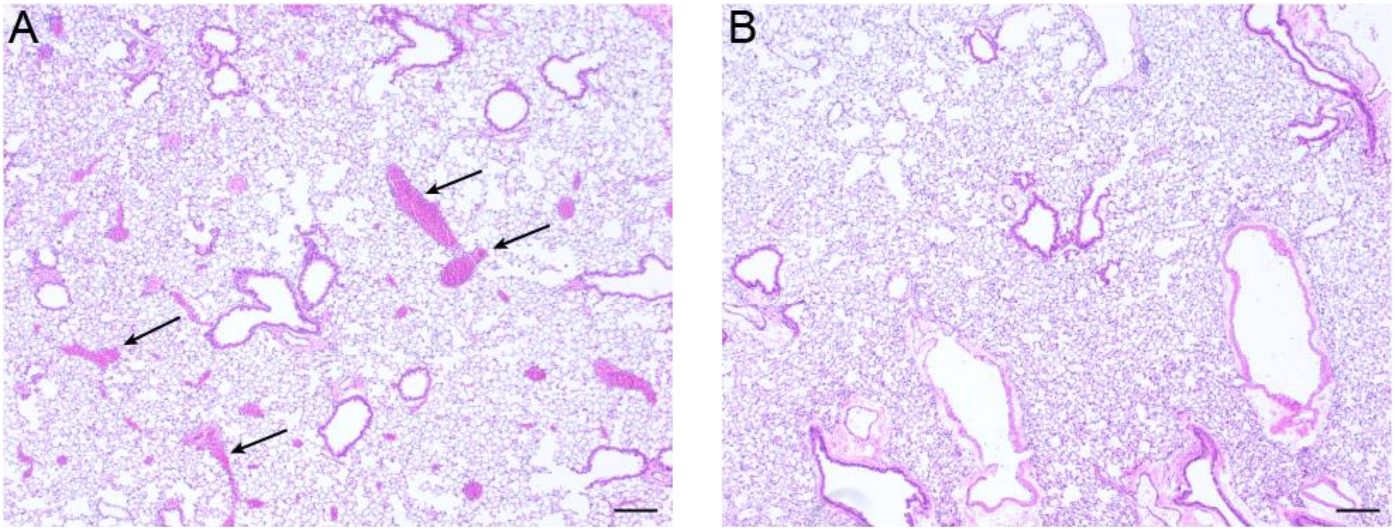

Supplementary Figure 1. Representative photomicrographs of H&E-stained sections of the lungs from **(A)** one control which had no intranasal fluid experience and **(B)** one experimental test subject that underwent intranasal cocaine self-administration. No significant histopathological abnormalities were noted in the lungs. The only difference amongst animals was slight to moderate vascular congestion (arrows) which was within normal limits. Scale bar = 200 µm.

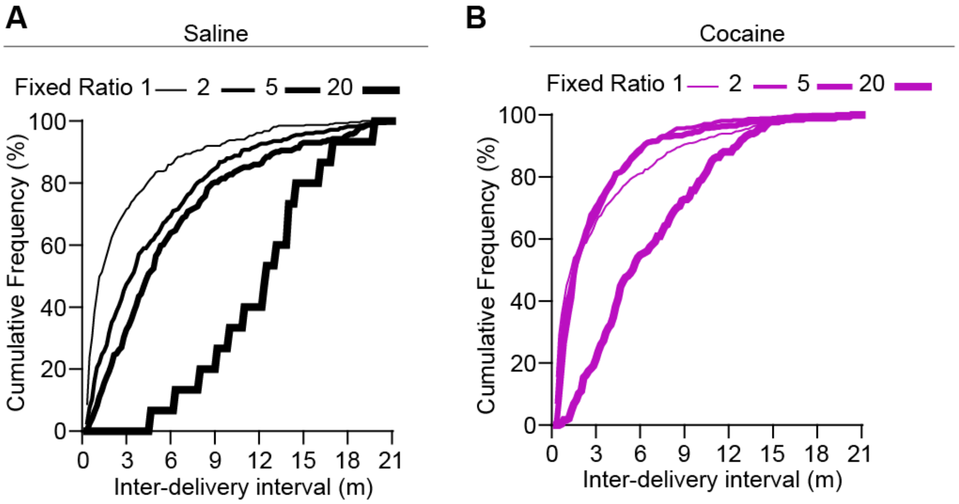

Supplementary Figure 2. Intranasal cocaine self-administration engenders a consistent inter-delivery interval across fixed ratio schedules. Cumulative frequency distributions of inter-delivery intervals during intranasal self-administration sessions for saline **(A)** and cocaine **(B)** groups across increasing fixed ratio. Each curve represents pooled inter-delivery intervals from all animals under a given fixed ratio schedule. In the saline group, increased ratio requirements lead to a rightward shift in the distribution, indicating increased inter-delivery intervals as a function of schedule (one-way ANOVA, $F_{(3, 496)} = 61.62$, $p < 0.0001$; Bonferroni post-test, fixed ratio 1 vs 2: $p = 0.0183$; fixed ratio 1 vs 5: $p < 0.0001$; fixed ratio 1 vs 20: $p < 0.0001$). In contrast, cocaine self-administration animals displayed consistent inter-delivery intervals which did not shift rightward until fixed ratio 20 (one-way ANOVA, $F_{(3, 496)} = 18.37$, $p < 0.0001$; Bonferroni post-test, fixed ratio 1 vs 2: $p = 0.99$; fixed ratio 1 vs 5: $p = 0.99$; fixed ratio 1 vs 20: $p < 0.0001$).
