# Supplementary material for: Intranasal cocaine self-administration in male mice
Source: Nat Commun. 2025 Dec 5;16:10916. doi: 10.1038/s41467-025-65875-w (PMC12680761; doi:10.1038/s41467-025-65875-w)
Supplement: Supplementary file 2 — Description of Additional Supplementary Files [file 41467_2025_65875_MOESM2_ESM.pdf]

Description of Additional Supplementary Files

Supplementary Video 1. Intranasal cocaine self-administration in a head-restrained mouse. Video clip of a mouse performing intranasal cocaine self-administration, played at 2x real-time. The subject was trained to press the lever on their right (on the left of the screen in the video) for cocaine under a fixed ratio 2 schedule of reinforcement. Completion of the ratio resulted in the immediate delivery of cocaine (dissolved in saline) directly in front of the subject's nostril. Note the flash in the video is the onset of the cue light presented concomitant with each cocaine delivery.
